# Energy Harvesting Materials and Structures for Smart Textile Applications: Recent Progress and Path Forward

**DOI:** 10.3390/s21186297

**Published:** 2021-09-20

**Authors:** Patricia I. Dolez

**Affiliations:** Department of Human Ecology, University of Alberta, Edmonton, AB T6G 2N1, Canada; pdolez@ualberta.ca

**Keywords:** smart textiles, energy harvesting, photovoltaic, piezoelectric, triboelectric, thermoelectric

## Abstract

A major challenge with current wearable electronics and e-textiles, including sensors, is power supply. As an alternative to batteries, energy can be harvested from various sources using garments or other textile products as a substrate. Four different energy-harvesting mechanisms relevant to smart textiles are described in this review. Photovoltaic energy harvesting technologies relevant to textile applications include the use of high efficiency flexible inorganic films, printable organic films, dye-sensitized solar cells, and photovoltaic fibers and filaments. In terms of piezoelectric systems, this article covers polymers, composites/nanocomposites, and piezoelectric nanogenerators. The latest developments for textile triboelectric energy harvesting comprise films/coatings, fibers/textiles, and triboelectric nanogenerators. Finally, thermoelectric energy harvesting applied to textiles can rely on inorganic and organic thermoelectric modules. The article ends with perspectives on the current challenges and possible strategies for further progress.

## 1. Introduction

With their ability to provide fabrics and garments with sensing, actuating, and/or adaptive functions in response to a wide range of stimuli, wearable electronics and smart/e-textiles have found a place in an increasing number of applications and commercial products. Textile-based sensors can detect pressure, strain, temperature, chemicals, humidity, obstacle, proximity, and location by converting the corresponding stimuli into electrical signals [1]. In the case of electrical stimuli, the signal can be directly accessed using electrodes. The largest number of sensor-type technologies, solutions, and products available respond to mechanical and electrical stimuli, with a proportion of 59% and 23%, respectively. One of the best know applications is for the monitoring of vital signs for athletes [2], patients [3], older adults [4], firefighters [5], and even astronauts [6]. In the case of textile-based actuators, thermal, optical, and power outputs each represent about 25% of the actuator-type technologies, solutions, and products currently available [1].

However, a major challenge with wearable electronics and e-textiles is the question of power supply [7]. Although a recent trend can be observed toward the development of low power devices [8,9,10,11], technologies currently available are relatively energy intensive. Most smart textile products are powered using traditional rechargeable batteries. However, these batteries are heavy and bulky; for instance, batteries account for about 9 kg or 20% of the carry-on load of soldiers on a mission [12]. In addition, because of their bulkiness, lack of flexibility, and inability to sustain laundering, they cannot be fully integrated within the textile architecture. There has been an effort to develop conformal and lightweight power generation and storage devices such as flexible or elastic batteries and supercapacitors [13]. Unfortunately, they have not reached the capacity of traditional batteries yet.

**Figure 1:**
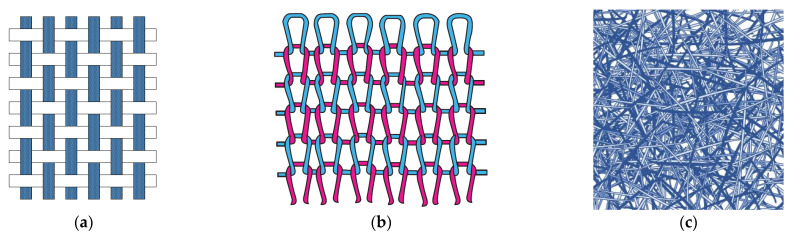
Examples of textile structures relevant to energy harvesting devices: (**a**) woven; (**b**) knit; (**c**) nonwoven mat (source: Md. Rashedul Islam, with permission).

As an alternative to batteries, energy can be harvested from various sources using garments or other textile products as a substrate [14]. Fabrics and clothing benefit from several advantages as an energy harvesting medium: they offer a large surface area and, in the case of clothing and many other applications; they are in intimate contact with the body as human’s closest environment. They also generally exhibit a good conformability to the complex deformations associated with body motions. They thus allow an easy access to the thermal and mechanical energy generated by the human body [15], as well as other sources of renewable energy such as the sun [14]. In addition, they are lightweight, flexible, stretchable, and breathable. With structures such as wovens (Figure 1a), knits (Figure 1b), and nonwovens (Figure 1c), along with the possibility to overlay additional components by embroidery for instance, they are an ideal platform for unobtrusive energy harvesting while preserving the comfort of the wearer.

The next sections discuss four different energy harvesting mechanisms relevant to smart textiles: photovoltaic, piezoelectric, triboelectric, and thermoelectric. In the case of photovoltaic energy harvesting technologies, new developments involve high efficiency flexible inorganic films, printable organic films, dye-sensitized solar cells, and photovoltaic fibers and filaments. Textile-based piezoelectric systems rely on polymers, composites/nanocomposites, and piezoelectric nanogenerators to collect the mechanical energy associated with deformations. For their part, textile triboelectric energy harvesting systems can take advantage of films/coatings, fibers/textiles, and triboelectric nanogenerators. Finally, inorganic and organic thermoelectric modules are used in thermoelectric energy harvesting applied to textiles. The last section of the article provides perspectives on the current challenges faced by textile energy harvesting systems and proposes possible strategies for further progress.

## 2. Photovoltaic Energy Harvesting

Clothing and other textile products such as backpacks and tents offer a promising opportunity to harvest indoor and outdoor light. For instance, values of the clothing area factor, which correspond to the ratio between the clothing surface area and the body surface area, have been reported to be between 1.05 and 1.31 for tight and loose fitting garments, respectively [16]. Body surface area averages 1.9 m^2^ in adult men, 1.6 m^2^ in adult women, and 1.07 m^2^ in 9-year-old children [17].

Photovoltaic systems function by converting the energy of incident light into electricity [18]. The photons are first absorbed by the photoactive material, which reaches an excited state. This leads to the generation of free positive- and negative-charge carriers. The transport of carriers of opposing charge in different directions and their final recombination complete the photovoltaic energy conversion process, which gives rise to the photocurrent. In addition to the photoactive layer(s) and the two electrodes, one of them being transparent, photovoltaic systems can include other layers such as hole and electron transporting layers and an antireflection coating, which are aimed at improving the photovoltaic cell performance. Photovoltaic cells rely on semi-conductors for the photoactive layer(s).

The thin film technology has allowed the production of flexible photovoltaic structures, giving textiles the potential to harness the energy of the sun while maintaining the conformability necessary to preserve the function of garments and other textile products [19]. Strategies include deposition of the photovoltaic thin film on a thin flexible steel or polymer substrate, which is then laminated to the textile, direct deposition of the photovoltaic thin film on a textile substrate, and incorporation of photovoltaic fiber/filaments/yarns into the textile structure. Technologies relying on polymer-based and dye-sensitized photovoltaic cells have also been developed; they have recently shown a large increase in power conversion efficiency.

### 2.1. High Efficiency Flexible Inorganic Photovoltaic Films

One of the leading technologies to produce high efficiency flexible photovoltaic films is based on the Cu (In,Ga)Se_2_ (CIGS) semiconductor compound [20]. Demonstrated efficiencies of up to 22.6% had been achieved by 2016 and researchers led by the Belgian institute imec announced in February 2020 that their CIGS-based flexible solar cell intended for integrated building applications reached an efficiency of 25% [21]. It is thought that an efficiency level close to 30% is technically feasible [20]. CIGC flexible photovoltaic film products are already commercially available, e.g., Solar Cloth System (www.solarclothsystem.com, accessed on 10 September 2021), Filsom (www.flisom.com, accessed on 10 September 2021), and PowerFilm Inc. (www.powerfilmsolar.com, accessed on 10 September 2021). For instance, they have been used for parking lot shades, tents, and sails [22]; the flexible inorganic photovoltaic film is bonded to a fabric substrate to provide a lightweight and flexible material.

Another strategy to produce high efficiency flexible inorganic photovoltaic films relies on amorphous silicon. For instance, a photovoltaic textile was prepared using a woven polyester fabric [23]. The lower electrode was formed by liquid-coating the fabric with a conducting conjugated polymer, poly(3,4-ethylenedioxythiophene) polystyrene sulfonate (PEDOT: PSS), and vacuum-evaporating a layer of aluminum on top of it; the role of PEDOT: PSS is to bridge eventual cracks in the aluminum layer when the fabric is flexed. Then, three layers of photoactive amorphous silicon, N-type/undoped/P-type, were deposited by plasma enhanced chemical vapor deposition (PECVD), which can be performed at a temperature compatible with polyester (200 °C). The top electrode was composed of sputtered transparent conducting oxide (TCO), which also acted as an encapsulation for the photovoltaic film. In another practical application of amorphous silicon, a photovoltaic rollable awning was designed using 50 μm-thick amorphous silicon-based thin film cells covered by a 50 μm thick transparent polytetrafluoroethylene protective layer and laminated on an acrylic awning fabric [24]. Bonding layers of 200 μm thick ethylene-vinyl acetate (EVA) were used between the different components of the flexible composite structure. No decline in performance was observed after one year of use, which included more than 2000 cycles of winding and unwinding.

In the case of the conventional semiconductor gallium arsenide (GaAs), a high-throughput epitaxial lift-off (ELO) process, developed to allow the reuse of the expensive GaAs substrates, opens the door to flexible applications [25]. After a thin film of GaAs has been grown on the single crystal GaAs wafer, it is lifted off from the substrate by surface tension created by immersing the wafer into hydrogen chloride up to the level of the etching front. The 1–3 μm GaAs thin film is then transferred to a flexible tape and sheet. Other researchers improved the traditional hydrofluoric acid (HF)-enabled ELO technique by using a HF-resistant p-ohmic contact based on AuBe/Pt/Au and coating the flexible substrate with a Cr/Au bilayer to facilitate the transfer from the GaAs substrate [26]. With this technique, they prepared a highly efficient single-junction GaAs thin-film solar cell on a flexible substrate that achieved an efficiency of more than 22%. In addition to the process of transfer by sacrificial layers corresponding to the ELO technique, the main transfer printing methods for the production of thin film solar cells include the transfer by porous Si layer, transfer by controlled crack, and transfer by water-assisted thin film delamination [27].

**Figure 2:**
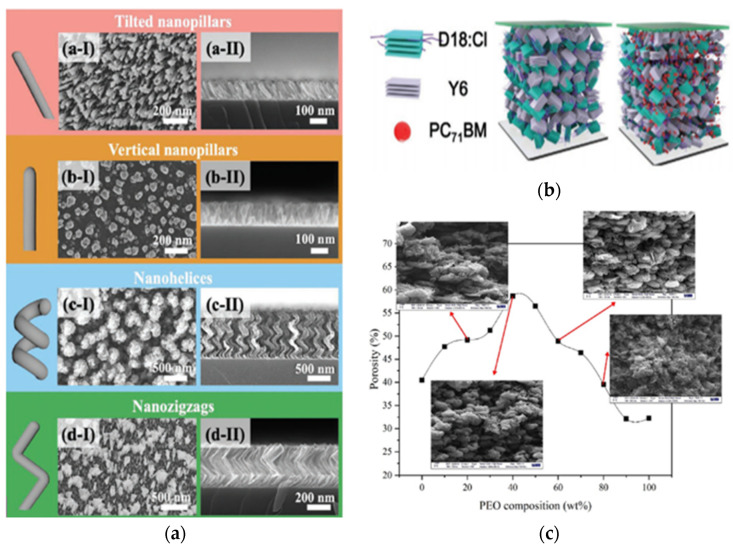
Examples of recent developments toward photovoltaic energy harvesting materials and structures for textile application: (**a**) different geometries of titanium nanopillars used as electron transporting layer in inorganic flexible solar cells (reproduced from [33] with permission from Wiley); (**b**) ternary polymer used as active layer in organic photovoltaic cells (reproduced from [36] with permission from Wiley); (**c**) porosity and morphology of PVDF-HFP/PEO microporous membrane used in gel-like dye-sensitized solar cells (reproduced from [37] with permission from Elsevier).

Recently, a new material has been presented as a game-changer for the thin-film photovoltaic industry [28]. Perovskites are a class of compounds that have the same type of crystal structure as calcium titanate (CaTiO_3_). Since the first attempt to use perovskite for solar cells in 2009, the conversion efficiency has increased from 3.8% to 25.2% in only 10 years. The stability of the perovskite organic–inorganic hybrid semiconductor has also been greatly improved by combining photoactive 3D and stable 2D perovskites [29]. In addition, the production process is relatively simple, the raw materials are available in large quantities, and the perovskite thin film can be made transparent [30]. However, most of the photovoltaic perovskites studied are lead-based. A recent improvement in the perovskite solar cell efficiency and resistance to moisture was obtained with the addition of 4-tert-butylpyridine (tBP) and the use of a larger iodine methylamine concentration, which led to the formation of larger, defect-free perovskite gains surrounded with hydrophobic tBP [31]. When researchers used graphene-based polyaniline nanocomposite instead of polyaniline as the hole transport layer, they observed an increase in the efficiency by more than 20% for inverted perovskite solar cells, in which the hole transport layer rather than the electron transport layer is deposited on the transparent electrode [32]. Their long-term stability also improved without the need for encapsulation. Another study reported that decorating a transparent electrode with a close-packed titanium nanopillar array (Figure 2a) passivated with a 5 nm thick titanium dioxide (TiO_2_) layer produced an electron-transporting layer with improved power conversion and long-term stability [33]. Perovskites have also shown great potential for the production of tandem structures with silicon and CICG [28]. However, concerns have been expressed regarding risks for health and the environment associated with lead-containing perovskite photovoltaic cells, which currently offer the best efficiency [34]. Yet, researchers recently showed that, with an expected operational lifetime of 20 years, the lead-containing perovskite photovoltaics technology has a four times lower lead intensity and 20 times lower toxicity potential compared to the current US grid electricity, which relies for a large part on coal power generation [35]. For instance, according to the researchers’ calculations, the amount of lead emission into the air, water, and ground associated with coal electricity production is at least as high as the amount of lead that would be contained in lead halide perovskite photovoltaic cells giving the same energy output.

### 2.2. Organic Photovoltaic Films

Polymer-based photovoltaic cells offer many advantages for smart textiles: they are flexible, thin and lightweight [38]. They can also withstand the deformations associated with a use in a garment. Another major advantage is that polymer photovoltaic cells can be prepared from liquid solutions using existing continuous processes such as roll-to-roll printing and coating with which the textile industry is already familiar [39].

For instance, textile organic solar cells were fabricated by dip coating using a stainless steel mesh fabric as a substrate and electrode [40]. Dip coating was employed to deposit the photoactive and hole transport layers while the metal back electrode was deposited by evaporation under vacuum. The power conversion efficiency reached 0.69%. Textile organic solar cells were also produced by spray coating [41]. An interface layer was first deposited by screen printing on the fabric to turn the rough woven structure into a smooth surface. Then, the different components of the photovoltaic cell were spray coated using a shadow mask: the Ag electrode, a zinc oxide (ZnO) layer, the active layer (PI-4), a PEDOT: PSS layer, and finally an Ag nanowire layer as the top electrode. A power conversion efficiency of 0.4% was obtained. A conversion efficiency of 10.30% was achieved by doping the PEDOT: PSS layer with a small amount of ethylene glycol [42]. The organic solar cell was prepared on a polyethylene terephthalate (PET) flexible substrate and also used Ag nanowires as the top electrode since this material has the advantage of flexibility and transparency, in addition to being easy to manufacture and low cost. The solar cell also showed remarkable mechanical performance; it retained 90% of its original conversion efficiency after 1000 bending cycles.

Roll-to-roll printing is another process for preparing polymer-based photovoltaic cells that can easily be implemented in the textile industry. For instance, a large-area, all-polymer solar cell was prepared using roll-to-roll slot die printing on a PET flexible substrate [43]. Power conversion efficiencies of 5% were obtained by carefully selecting the donor and acceptor polymer pairs to reduce the crystallinity of conjugated polymers in the bulk heterojunction active layer. Demonstration of the applicability of the process to textile substrates includes organic solar cells prepared on a flexible, transparent and conductive woven fabric electrode using poly(3-hexylthiophene) (P3HT) and 1-(3-methoxycarbonyl)-propyl-1-phenyl-(6,6) C61 (PCBM) [44]. The power conversion efficiency obtained was 3.1%.

Inkjet printing has also been reported as a promising process to prepare organic solar cells for flexible applications [45]. Its advantages include low-cost manufacturing, low material waste, the possibility of large area formation and precise localization, and large process flexibility. However, challenges exist such as limitations in terms of solution viscosity, nozzle clogging, and the formation of a ring-like deposit once the solvent evaporates (coffee-ring effect).

Recent progress in terms of power conversion efficiency was made through ternary polymerization and side-chain engineering. For instance, a power conversion efficiency of 13.26% was obtained by adding 30% of a non-fluorinated component BDTPS into a fluorinated polymer o-PBTA-PSF composed of donor and acceptor units [46]. Through random copolymerization, the ternary polymer offers a weakened conformational locking, leading to a reduction in the aggregation behavior. In another study, the addition of the amorphous PC_71_BM molecule to D18-Cl and Y6 (Figure 2b) reduces the crystallization and amount of aggregates [36]. The power conversion efficiency of the ternary organic solar cell is 15.5% and it offers an unprecedented power-per-weight ratio of 32 W g^−1^. The active layer is also more flexible and less brittle, and the device displays an excellent long-term durability. Using sidechain engineering, researchers added alkoxyl chains to the two outermost thiophene rings of an electron acceptor material, affecting the molecular orientation, crystallinity, and morphology of the photoactive layer and leading to a power conversion efficiency of up to 12.65% [47].

Recent work also involved looking for higher transmittance alternative to the PET flexible substrate used in PEDOT: PSS solar cells [48]. Power conversion efficiencies of 15.3% under sun light and 20.5% under indoor light were achieved using a surface-textured polydimethylsiloxane substrate. The solar cell system also displays a lower sensitivity to light orientation compared to its indium tin oxide (ITO) counterpart.

### 2.3. Dye-Sensitized Solar Cells

Dye-sensitized solar cells are the least expensive because of the simplicity of the implementation and low cost of the raw materials and fabrication equipment [49]. As they are highly flexible, they are a great candidate for smart textile applications. The textile industry is also highly familiar with the use of dyes. For instance, a textile-based dye-sensitized solar cell was prepared on a fiberglass woven fabric to provide the needed thermal stability during the manufacturing [50]. A thin layer of polyamide was first applied on the fabric by roll-to-roll before the layers corresponding to the different components of the dye-sensitized solar cell were deposited. The conversion efficiency was 1.8% at 20% of the sun intensity. No change in efficiency was observed over a 7-week period.

To accommodate for the low temperature resistance of most fabrics, a low temperature TiO_2_ paste was used to prepare a dye-sensitized solar cell on a 65/35 polyester cotton woven fabric [51]). The fabrication method involved screen printing and spray coating. A conversion efficiency of 2.87% was obtained. The same authors reached a conversion efficiency of 3.24% on the same polyester cotton fabric when they used flexible polyethylene terephthalate/indium tin oxide (PET/ITO) as the counter electrode instead of the conventional fluorine doped tin oxide (FTO) glass [52].

Other recent improvements involved the development of new isomeric ruthenium complex sensitizers with an imine-carbene-based ligand [53]. When used pure, they led to conversion efficiencies of up to 6.59%. Gel-like dye-sensitized solar cells have also been designed to overcome the challenges associated with volatile liquid electrolytes. For instance, an ionic liquid electrolyte containing dispersed hydrophilic cobalt-functionalized nitrogen-enriched graphene oxide sheets showed a power conversion efficiency of 7.3% [54]. Other quasi-solid-state dye-sensitized solar cells were prepared using poly(vinylidene fluoride-co-hexafluoro propylene) copolymer and polyethylene oxide (PVDF-HFP/PEO) as a polymer matrix (Figure 2c) and various imidazolium-based ionic liquids [37]. The highest ionic conductivity sample offered a power conversion efficiency of 6.47% under sun exposure. The long-term durability was improved compared to solar cells using a liquid electrolyte.

Progress has also been made in the area of electrodes with the use of nanofibrous transparent electrodes [55]. They were prepared by applying with a brush a PEDOT: PSS solution in polystyrene sulfonate on PVDF nanofibers. The resulting electrode displayed a transmittance of 84% at a wavelength of 550 nm as well as a sheet resistance of about 1.5 kΩ/sq.

### 2.4. Photovoltaic Fibers/Filaments

Photovoltaic fibers/filaments can be produced by yarn slitting from an ordinary solar film, monofilament spinning with electrode/photovoltaic materials/transparent counter electrode coatings, or conjugate spinning of conductive polymer/organic semi-conductor/TiO_2_ protective layer followed with coating by transparent electrode material [56]. The fibers/filaments can then be made into textiles using weaving, knitting, braiding, and embroidery for instance.

For instance, researchers have taken advantage of the different layers formed when weaving a fabric to create a 100% textile dye-sensitized solar cell [57]. The active components were positioned in the weft direction: a dye-loaded, TiO_2_-coated holed metal ribbon as photoanode and a Pt nanoparticle-loaded carbon yarn as a counterelectrode, separated from each other by polyamide warp yarns. Two metal wires woven in the warp direction and positioned at each extremity of the photoanode ribbon and in electrical contact with it served as power outlets. The textile solar cell was prepared using a standard loom. An energy conversion efficiency of 2.63% was obtained. Fiber-shaped organic photovoltaic cells were produced with an inverted polymer-fullerene bulk-heterojunction structure and woven into a textile [58]. The resulting fabric displayed excellent flexibility, folding properties, and electric performance. Progress has also been achieved toward the production of perovskite solar fibers that may eventually be integrated into a textile structure [59]. The fiber configurations used are mostly of the core-shell type, but also include double-twisting, helical filament wrapping, and nanorod protrusions. However, challenges remain, for instance the sensitivity to chemical and mechanical degradation, in particular with bending, lack of flexibility, high annealing temperature required, material toxicity, limited surface area available to light exposure in the case of a unidirectional light source, and low power conversion efficiency. Another strategy involves embedding tiny silicon solar cells connected with thin copper wires within the fibers of a textile yarn [60]. The yarns were then woven to produce a fabric containing 200 miniature solar cells. A 20 cm^2^ piece of the fabric was able to power a basic mobile phone under sun exposure. The fabric also maintained 90% of its efficiency after 15 machine washes.

**Figure 3:**
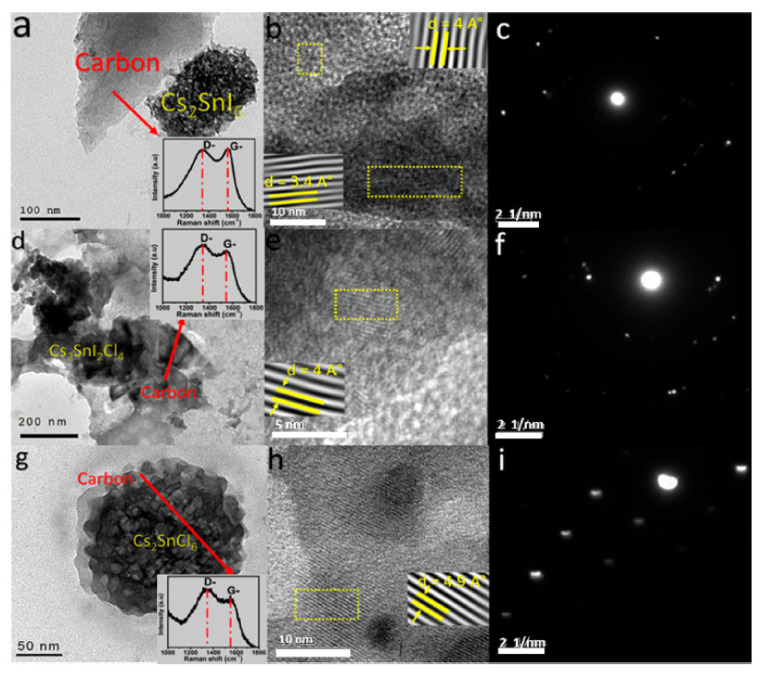
Examples of recent developments toward photovoltaic energy harvesting materials and structures for textile application: image by high-resolution transmission electron microscopy of double perovskites after annealing: Cs_2_SnI_6_ (**a**–**c**), Cs_2_SnI_2_Cl_4_ (**d**–**f**), Cs_2_SnCl_6_ (**g**–**i**), with Raman spectra of the graphene oxide nanoparticles formed shown in inserts in a, d, and e (reproduced from [62], with permission from ACS Publications).

A strategy explored by researchers to increase the level of conversion relies on electrospun fibers [61]. Electrospinning allows preparing one dimensional random or oriented fibrous mesostructures using organic, inorganic and hybrid nanomaterials. Core–sheath, hollow, and porous morphologies can be created, even multichanneled microtube arrangements. The gain in energy conversion performance comes from the nanoscale features of the electrospun nanofiber mats: high surface area, large aspect ratio, low density, and large porosity. For example, single-phase electrospun double perovskite nanofibers were prepared using iodide and chloride [62]. The resulting material is lead-free, which provides an answer to the concerns in terms of toxicity associated with perovskites. A layer of graphene oxide formed during the annealing process (Figure 3), which decreased the bandgap energy. The best results were obtained for perovskites with a mix iodide and chloride ions.

New developments with the counter electrode for a fiber solar cell configuration was made using a carbon nanotube yarn [63]. A slight increase in the power conversion efficiency was obtained with 4% as compared to 2.64% with platinum as a representative to the noble metals traditionally used.

## 3. Piezoelectric Energy Harvesting

Of the three main ways to transform mechanical energy into electrical energy, piezoelectric systems, which rely on the electromechanical interaction between materials mechanical and electrical states, are the most widely studied [64]. They are the most relevant when the application requirements include high voltage, high energy density, high capacitance, and low mechanical damping. Materials exhibiting piezoelectricity can be grouped in four categories: single crystals, ceramics, polymers, and polymer composites/nanocomposites. However, several of them are brittle, rigid, and/or toxic. Polymers such as polyvinylidene fluoride as well as composites and nanocomposites appear to offer the best perspectives for smart textile application. New developments relevant to smart textiles include also piezoelectric nanogenerators, i.e., energy harvesting systems taking advantage of small mechanical deformations for energy generation.

A patent describes a fabric-based piezoelectric energy harvesting device [65]. It involves linear or serpentine piezoelectric harvesters connected to the fabric and positioned at different strategic locations (e.g., elbows, shoulders) on the garment. The patent also describes energy storage media.

### 3.1. Polymers

Polyvinylidene fluoride (PVDF) is by far the most studied polymer for application in smart textiles. It can be used as a film and embedded into a textile. For instance, shell structures formed of a PVDF film fixed with a two-side adhesive on a polyester film were inserted into gussets in a fabric [66]. The best results were obtained with the polyester film being curved. A maximum output voltage of 25 V was measured when the fabric was secured around a bending elbow. PVDF can also be made into multifilament yarns by melt-spinning and then woven into 100% PVDF 2D and 3D structures [67]. The 3D interlock structure provided an output voltage of 2.3 V when a 5 N compression force was applied perpendicularly to the fabric plan using a dynamic mechanical analyzer. By comparison, the 2D plain weave fabric only provided 0.14 V when loaded in the same conditions. Another strategy explored by researchers involves electrospun fibers. In particular, experiments involving multilayer electrospun PVDF mats with different fiber alignments and layering angles showed that the best performances are obtained with well-aligned fibers and layering angles of 120° from the fiber direction of the base layer [68]. The improvement in the electrical response when the mats were subjected to a periodic mechanical impact was equal to 95% in the first case and 41% in the second case. The electrospinning parameters can be optimized to increase the fraction of β phase and crystallinity, thus improving the piezoelectric performance of the PVDF nanofibers [69].

**Figure 4:**
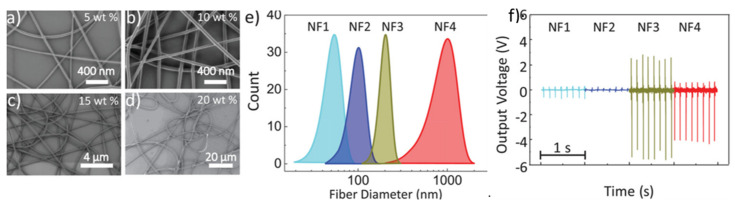
Examples of recent developments toward piezoelectric energy harvesting materials and structures for textile application: (**a**–**d**) SEM images of piezoelectric electrospun PVDF fibers of different diameters (**e**) exhibiting different open circuit output voltages (**f**) (reproduced from [73], available through the Creative Commons license (creativecommons.org/licenses/by/4.0/)).

Other polymers exhibiting interesting piezoelectric properties include polylactic acid (PLA), cellulose, polyamides, polyurea, and polyurethanes [70]. For instance, poly(L-lactic acid) (PLLA) exhibits shear piezoelectricity when the PLLA molecules are oriented uniaxially by drawing or elongation and crystallized by heat treatment. Piezoelectric PLLA fibers can be prepared by micro slitting the piezoelectric PLLA film or directly by melt spinning [71]. A fabric was knitted with SZ yarns made by combining left-handed (S-yarn) and right-handed (Z-yarn) helical multi fiber yarns. The fabric displayed piezoelectric properties when extended and released. High shear piezoelectricity was also obtained with nanofibers prepared by electrospinning poly(D-lactic acid) (PDLA), PLLA, and carbon quantum dots (CDQ) [72]. The stereocomplex PDLA/PLLA/CQD nanofibers were subjected to more than 10,000 consecutive cycles without showing a decrease in the electrical output. Recently, researchers achieved the production of piezoelectric polyamide 11 fibers (Figure 4) by electrospinning the polymer in a low boiling point solvent, trifluoroacetic acid, in the presence of acetone [73]. The rapid evaporation of the solvent led to the formation of the piezoelectric phase δ’. The maximum voltage of 6V under periodic impact at a frequency of 8 Hz was obtained with 200 nm fibers.

Certain experiments have also been conducted using a natural polymer, namely cellulose. The piezoelectric characteristic of cellulose was evidenced 50 years ago with wood [74]. Recently, researchers have achieved peak-to-peak voltage of 1.2 V by applying a 20 N force at a frequency of 7 Hz using a mercerized cotton fabric sandwiched between two aluminum electrodes [75]. The output increased to about 47 V when a larger pressure was applied using finger tapping. To increase the efficiency of the device, they positioned a polypropylene tape on each side of the aluminum, cotton fabric, and aluminum assembly to create a piezoelectric-cum-triboelectric energy harvester, and reached an output voltage of 1.77 V.

### 3.2. Composites/Nanocomposites

A core-sheath piezoelectric microfilament with a carbon black/polyethylene conducting core and a PVDF sheath was prepared by melt-spinning [76]. Yarns were produced with 24 bicomponent filaments and used as a warp yarn to weave a piezoelectric textile. Conductive yarns were inserted in the weft direction and served as the outer electrode. As the inner electrode is completely shielded by the PVDF sheath, this fabric can operate in wet conditions. With a polyamide/stainless steel staple yarn or a silver plated polyamide yarn, the woven fabric generated 3V when subjected to strains of 0.25%. PVDF was also combined with piezoelectric barium titanate (BaTiO_3_) nanoparticles to form nanocomposite piezoelectric filaments by melt-spinning [77]. The filaments were then used to form piezoelectric textile structures using triaxial braiding, weaving, and circular knitting. The best performance in terms of power and sensitivity was obtained with the knitted fabric. In another study, the BaTiO_3_ nanoparticles were combined with a copolymer of PVDF, poly(vinylidene fluoride-co-trifluoroethylene (PVDF-TrFE) [78]. The nanoparticles were coated with a thin layer of PVDF-TrFE to facilitate their dispersion in the polymer matrix. The BaTiO_3_/PVDF-TrFE nanocomposite film exhibited an output voltage of 59.5 V under a 100 N load at a frequency of 2.5 Hz. When applied by dip-coating on a stainless steel wire spring, with a silver yarn wrapped around as the top electrode, an output of 1.66 V was measured under a 10 N compressive force at 2 Hz. A tricomponent system was also produced by combining PVDF, BaTiO_3_ nanoparticles, and reduced graphene oxide (rGO) nanoplates [79]. Nanocomposite fibers manufactured by melt-spinning were shaped into a coiled structure by twisting and coiling. They could be deformed up to 100%, which led to a peak output of about 1.3V with an energy conversion efficiency of 22.5%. The peak power density obtained is 3 W kg^−1^, which the authors of the study mentioned is 2.5 times more than what had been previously reported for piezoelectric textile structures. Prototypes of energy harvesting systems were produced by knitting and weaving to demonstrate the promising potential of the coiling strategy.

**Figure 5:**
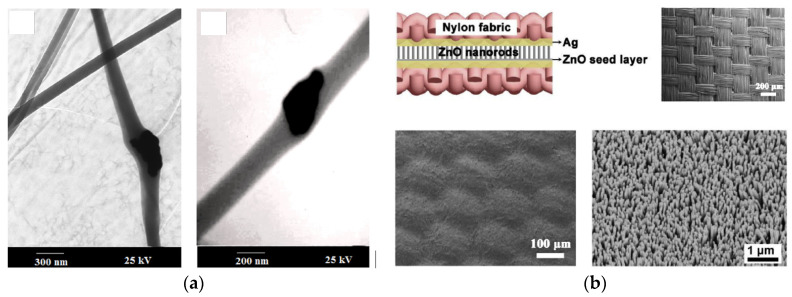
Examples of recent developments toward piezoelectric energy harvesting materials and structures for textile application: (**a**) PZT-PVDF nanocomposite nanofibers (reproduced from [80] with permission from Elsevier); (**b**) array of vertical ZnO nanorods on a silver coated polyamide fabric (top left cartoon: PENG structure, top right picture: uncoated woven fabric, bottom left picture: silver-coated fabric, bottom right picture: ZnO nanorods-coated fabric, reproduced from [83] with permission from Elsevier).

PVDF nanocomposite nanofibers have also been produced for piezoelectric energy harvesting. For instance, PVDF was associated with lead zirconate zitanate (PZT), a piezoceramic, in the form of nanocomposite nanofibers loaded with PZT nanoparticles (Figure 5a) [80]. The nanocomposite nanofibers were then used to prepare a flexible piezoelectric energy harvesting device. An output voltage of 184 mV was obtained under an applied force of 2.125 N. PVDF nanocomposite nanofibers were manufactured with graphene-ZnO nanocomposite nanoparticles [81]. This led to an increase in the nanofiber crystallinity and β phase content. The piezoelectric response reached a peak-to-peak voltage of 840 mV under a 1N applied force. Efforts have also been made to develop lead-free alternative piezoceramic fillers for more health- and environmentally friendly strategies [82].

In addition to PVDF-based composite and nanocomposite, other materials have been investigated for textile applications. For instance, piezoelectric textile nanocomposite multifilament yarns were prepared by melt extrusion and drawing of polypropylene with multiwalled carbon nanotubes [84]. If the performances displayed by the yarn are still low, polypropylene offers the advantages of high mechanical strength, good chemical resistance, and low cost. A printable piezoelectric paste was obtained by mixing two different sizes of PZT microparticles with silver nanoparticles and a polymer binder dissolved in a solvent [85]. It was applied on polyester–cotton, cotton, and polyamide–imide fabrics by screen printing. A layer of UV-curable polyurethane was used as an interface to compensate for the fabric roughness before depositing the silver polymer ink serving as the bottom electrode, followed by the PZT/silver nanoparticle active layer, and the silver polymer ink serving as the top electrode. Cold isostatic pressing was applied to reduce the voids in the nanocomposite layer. The performance in terms of energy density obtained when the fabrics were subjected to compression and bending deformation increased with the addition of the silver nanoparticles and the cold isostatic pressing treatment. When loaded in compression, the best results were obtained with the most compliant fabric while the fabric with the highest Young modulus performed the best in bending. Finally, piezoelectrets, which display a high piezoelectric activity when deformed after being charged, offer an alternative to conventional piezoelectric materials. Polydimethylsiloxane (PDMS) was combined with ZnO nanoparticles and spun cast on both sides of a silk textile [86]. A peak voltage of close to 3 V was obtained with the application of a 200 N force.

### 3.3. Piezoelectric Nanogenerators

Various designs have been proposed for piezoelectric nanogenerators (PENG) with the level of miniaturization desired for smart textiles and the level of actuation associated with human motion. For instance, hybrid piezoelectric fibers were produced with aligned BaTiO_3_ nanowires and a polyvinyl chloride (PVC) polymer [87]. These fibers were used in the warp direction to prepare a plain weave fabric, with copper wires and cotton yarns inserted in the weft direction as interdigited electrodes and insulating spacers, respectively. The cotton yarns also provided strength to the fabric. Two copper wires positioned along the warp direction on each side of the fabric and connected to the interdigited electrodes served as extraction electrodes. Finally, the nanogenerator fabric was poled at 70 °C to create the dipole moment along the active fibers. When used as part of an elbow band, it generated voltages of close to 2 V and an output power of 10 nW. Improvements in the performance can be achieved by optimizing the nanogenerator design. PVDF electrospun nanofibers have also been used to prepare yarns by twisting and plying [88]. These yarns were then woven into fabrics. The researchers showed that the piezoelectric response of the fabric improved for low yarn linear density and when increasing the yarn twist, number of plies, and fabric density. They achieved a voltage of 2.5 V under a 280 mN force. A finite element model of a piezoelectric nanofiber nanogenerator integrated foam corresponding to an application as a shoe insole was developed and experimentally validated with cyclic impact tests simulating a walking motion [89]. The model shows that a voltage of 15.1 V can be achieved with a nanofiber PENG-integrated foam with a modulus of 211 kPa.

ZnO nanorods patterned on textiles have also been explored to prepare textile-based PENG. A new hydrothermal method was developed to synthesize an array of uniform, densely packed, and vertically arranged ZnO nanorods on the silver coated surface of a polyamide woven fabric serving as one of the PENG electrode (Figure 5b) [83]. Another piece of the same fabric, also with the screen printed silver coating, was used as the counter electrode to complete the sandwich structure. Power outputs of 80 nW and 4 nW were obtained with palm clapping and finger bending, respectively. Several light-emitting diodes (LEDs) were lighted by foot stepping actuation. Silver doping of ZnO nanorods grown on a cotton fabric increased by a factor of three the output power of the PENG formed with the patterned fabric sandwiched between two thin copper sheets [90]. A force of 29 N generated an output power density of 1.45 mW cm^−2^. In another study, ZnO nanorods were grown on a Cu/Ni coated polyester woven fabric [91]. They were coated with copper thiocyanate to passivate their surface. Then a layer of PEDOT: PSS was added to form a p-n junction. Gold was evaporated on top of the PEDOT: PSS layer to form the top electrode. Finally, the entire system was encapsulated with PDMS. The device was tested using a permanent magnetic shaker at a 26 Hz frequency. The output voltage raised from 0.4 to more than 1.2 V when the ZnO nanorods length was increased from 2.3 to 5.9 μm.

A hybrid textile nanogenerator was prepared with cascaded piezoelectric and triboelectric units [92]. The piezoelectric unit was formed with electrospun nanofibers of PVDF/carbon nanotube/BaTiO_3_ particles enclosed between two layers of conductive fabrics. The polydimethylsiloxane-based triboelectric unit was positioned on top of the piezoelectric unit. A 4.5 × 5 cm prototype of the hybrid textile nanogenerator generated a power output of 2.22 W m^−2^, sufficient to drive 150 LEDs.

## 4. Triboelectric Energy Harvesting

Compared with the other mechanical energy harvesting processes, triboelectric energy harvesting, which relies on rubbing contact and electron transfer [93], offers many advantages [64]. This includes high power density, high conversion efficiency, and flexibility of the device, which makes it perfect for textile applications. However, there are still challenges to be solved, in particular regarding reliability and durability.

Combinations of materials used in triboelectric energy harvesting devices for textile applications include PDMS with latex or aluminum, polytetrafluoroethylene (PTFE) with polyamide 6 fabric or carbon nanotubes on a cotton fabric, fluorinated ethylene propylene (FEP) with latex or polyamide 6 fabric, polyimide (PI) with cotton fabric (denim), and polyester with polyamide 6 fabric [94]. They may be applied as laminated films or coatings to textiles or be directly in a textile form. They may also be part of a textile triboelectric nanogenerator (TENG), which describes energy harvesting systems taking advantage of small displacements for energy generation.

### 4.1. Films/Coatings

**Figure 6:**
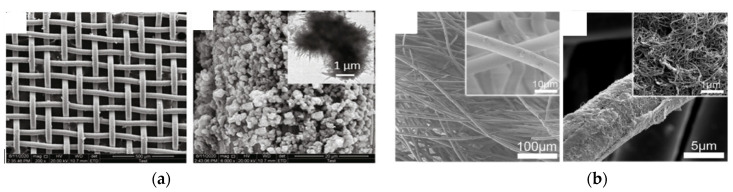
Examples of recent developments toward triboelectric energy harvesting materials and structures for textile application: (**a**) TiO_2_ flowers decorating a stainless steel textile (reproduced from [96] with permission from Elsevier); (**b**) CNT decorating a velvet fabric (reproduced from [100] with permission from ACS Publications).

Fully elastomeric triboelectric energy harvester devices were prepared by film casting and stencil printing using stretchable PDMS on one side and flexible but non-stretchable polyurethane (PU) on the other side [95]. Both materials had microbumps on the side on which they faced each other. Carbon-loaded versions of the two elastomers positioned on the outside of the active layers served as power outlets. Two configurations were prepared, a sliding mode and a vertical design (contact separation mode). The sliding mode provided the highest performance, with 3.6 μW cm^−2^. In another study, electrophoretic deposition was used to form microscale TiO_2_ flower-like features on the surface of a stainless steel woven fabric (Figure 6a) [96]. The flower-decorated fabric coated with PDMS was tested against cotton and silk cloth in contact–separation mode. Output voltages and currents of 120 V and 25 μA and 110 V and 30 μA were obtained with the cotton and silk cloth, respectively. The triboelectric system sustained 8000 cycles without a loss in performance. Another configuration involves alternating grated strips of positive and negative triboelectric materials on one side and screen-printed interdigitated Ag electrodes on the other side [97]. Power is generated in the sliding mode. The alternating grated striped surface was prepared by heat transfer of polyvinylchloride (PVC) on a polyamide fabric. A voltage of 136 V was generated. Fabric patterning was also created with 1.4 mm high embroidered protrusions on a surface of a woven conductive fabric made with stainless steel fiber/polyester filament spun yarn [98]. The same stainless steel fiber/polyester filament spun yarn was used to form the protrusions. PDMS served as the counter surface. A strong increase in the output was observed with the protrusions. The increase varied with the geometrical characteristics of the embroidered pattern.

Nanopatterning was achieved by applying carbon nanotubes (CNT) dispersed in PDMS on a silver textile using a brush [99]. The other side of the silver textile was covered with a copper layer. When tested against a polyamide cloth, the output voltage and the current increased by more than 3.5 times compared to the traditional dip-coating method. Hierarchical structures were produced on a velvet fabric through chemical grafting of CNT and poly(ethylenimine) (Figure 6b) [100]. This led to a 10-time increase in the output voltage and current, with a power density of up to 3.2 W m^−2^ when tested against a PTFE film/fabric. A triboelectric system for clothing application was also designed by combining a polyamide woven fabric, an e-PTFE membrane, and a polyurethane knitted fabric [101]. Gold nanodots were formed on the polyurethane surface by metal deposition and plasma etching to provide power generation through in-plane sliding motion. The maximum output power was about 2 μW with a cotton fabric.

Two strategies have been proposed to overcome the issue of moisture sensitivity usually observed with triboelectric energy harvesters. The self-assembled monolayer (SAM) technique was used to increase the hydrophobicity of substrate fabrics [102]. The coating materials were also selected to provide the required difference in electronegativity for triboelectric power generation: 1H,1H,2H,2H-fluorooctyl triethoxysilane (FOTS) was deposited on a polyester fabric and octadecyltrichlorosilane (OTS) or trichloro(3,3,3-trifluoropropyl) silane (TFPS) was used for the nickel-coated polyester fabric used as the opposing surface. The system was tested in vertical compression mode and showed an improved preservation of the energy-generating ability in high relative humidity environments of up to 85% relative humidity (RH). In another work, multiwall CNT were combined with a thermoplastic elastomer (TPE) and spray-coated on a fabric [103]. A micro-/nanostructured superhydrophobic surface was then created by ethanol etching. The counter surface was prepared with silicone rubber (EcoFlex) using the micro/nanostructured surface as a mold. The CNT loading greatly increased the power generating efficiency of the system, and slightly improved the performance in high humidity environments.

### 4.2. Fibers/Textiles

**Figure 7:**
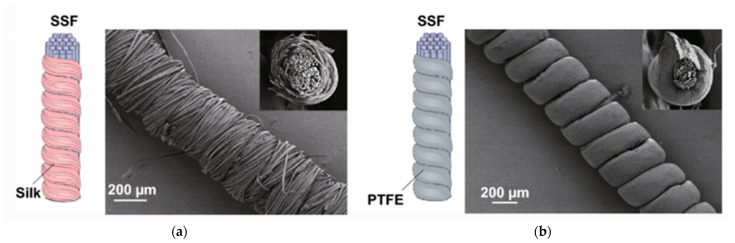
Example of recent developments toward triboelectric energy harvesting materials and structures for textile application: core-spun yarns composed of silk (**a**) and PTFE (**b**) fibers wrapped around a stainless steel fiber (SSF) core (reproduced from [104], available through the Creative Commons license (creativecommons.org/licenses/by/4.0/).

A silk-based triboelectric energy harvesting system was developed with the aim of providing an optimal trade-off between triboelectric and mechanical performance while preserving the flexibility for textile applications [104]. It is based on core-spun yarns (Figure 7). The electropositive side involves a stainless steel fiber core and a silk fiber wrapped sheath while the electronegative side is composed of a stainless steel fiber core and a PTFE fiber wrapped sheath. Two types of textile structures were produced using weaving and embroidery. These processes are easily scalable to production and allowed producing triboelectric energy harvesting devices with high strength, good energy generation output and great durability. A similar configuration of core-spun yarns produced with a stainless steel core and polyurethane wrapped sheath woven into a fabric was assessed with a series of typical clothing materials—polyamide, cotton, wool, polyester, and polyacrylonitrile—and exhibited output voltages between 20 and 80 V [105]. PVDF fibers were also electrospun on a CNT yarn core acting as the electrode [106]. When tested against a polyamide counter material on an electrode in contact–separation mode at 2 Hz and 10 N to simulate a heel strike, the PVDF-CNT coaxial yarn provided an output of 20 μW cm^−2^. The yarn exhibited a good durability; the output power increased by about 30% after 200,000 testing cycles and remained globally unaffected after 10 washing cycles. The yarn also resisted to more than 1000 rubbing cycles against a steel ball under an applied stress of 5MPa. Another strategy involves preparing a 100-m long polypropylene-clad tungsten fiber by thermal drawing and weaving it into a fabric [107]. It was tested for triboelectric power generation in the compression mode using a polyamide fabric and was able to power 62 LEDs. The durability over 100,000 cycles and washability were also assessed.

In another study, fabrics composed of natural fibers, cotton and silk were used to create piezoelectric energy harvesting systems by dip-coating them in cyanoalkyl silane for a positive triboelectric characteristic and fluoroalkyl silane for the negative triboelectric characteristic [108]. The output voltage and current achieved were 216.8 V and 50.3 μA. Wood-based cellulose in different forms has also been used for its own piezoelectric performance [109]. This includes cellulose nanofibers, cellulose nanocrystals, cellulose aerogels, and lignin in combination with various organic and inorganic compounds. Recent efforts have aimed at increasing the triboelectric charge density of the wood cellulose-based devices by chemical modification and effective contact area improvement.

**Figure 8:**
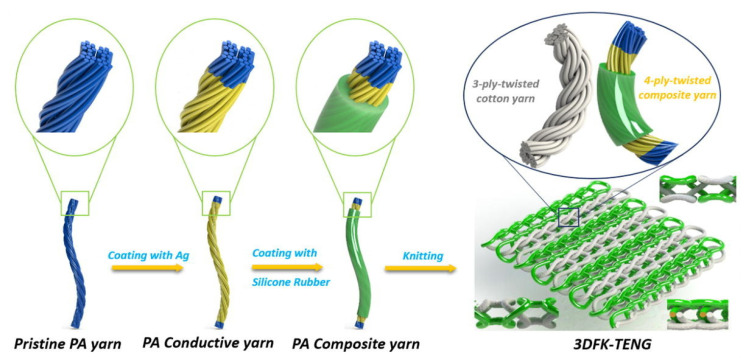
Example of recent developments toward triboelectric energy harvesting materials and structures for textile application: 3D TENG composed of a double-faced interlock knit containing polyamide (PA) 66 yarns coated with silver and silicone rubber and cotton yarns (reproduced from [112], with permission from Elsevier).

Stretchable textiles are desirable for deformation-based piezoelectric systems. A highly stretchable piezoelectric knit fabric was created using a rib stitch structure with a double twisted yarn comprised of cotton threads and lacquered copper wires [110]. Alternating rows were created using the yarn coated with polyamide and coated with PVC as the positive and negative components of the energy harvesting system. A 29 V output was produced when a 100% strain was applied to the knitted fabric. No changes were recorded after 60 washing cycles. Stretchable fabric structures were also prepared using a negative Poisson’s ratio yarn [111]. A multifilament polyamide yarn coated with silver was helically wrapped around an elastic core using ring spinning to produce the negative Poisson’s ratio yarn. The piezoelectric energy harvesting relies on the polyamide and rubber components contacting and separating when the negative Poisson’s ratio yarn is stretched. A one-component triboelectric structure was also prepared using 3D double-faced interlock knitting [112]. Alternative ribs were formed with a cotton yarn and a composite yarn composed of a polyamide 66 yarn first coated with silver and then with silicone rubber (Figure 8). The fabric generated 40V when loaded in compression and 2V when stretched.

Double-faced knitted fabrics combining the conductive component on one side and the piezoactive component on the other side were produced using the plating stitch technique [113]. The conductive face was composed of a silver-coated polyamide yarn and the piezoactive face involved dielectric yarns such as polyamide, polyethylene, PTFE, and polyester. In a coplanar sliding mode at 3 Hz with polyethylene and polyamide 66 as the active yarns, the output voltage was 120 V and the current about 1.6 μA, lighting up 200 LEDs. In a contact–separation mode also at 3 Hz, polyamide 66 and PTFE as active yarns led to an output voltage of 232 V and a current of 6.8 μA, lighting up 250 LEDs. The fabrics displayed a high air permeability and low bending stiffness, indicative of a high comfort. Large efficiencies when tested in walking and hand clapping scenarios were also achieved with a stainless steel fiber fabric and a layer of foam secured on a copper substrate [114]. Samples of 9 × 9 and 14 × 21 cm^2^ successfully lit 52 and 190 LEDs, respectively. The advantage of this design lies in the use of low-cost materials and fabrication techniques. High outputs were obtained with a rubber-based woven structure combining three charge flow paths: internal triboelectric effect with rubber against Ag-coated glass microspheres loaded rubber, interlaminar triboelectric effect with PTFE powder-loaded rubber against Ag-coated glass microspheres loaded rubber, and external triboelectric effect with aluminum against PTFE powder-loaded rubber and PDMS [115]. The global output in contact–separation mode under 50 N at 3 Hz reached 728 V and 16.6 μA.

### 4.3. Textile Triboelectric Nanogenerators

A fiber-to-fiber TENG was developed using ethyl cellulose co-electrospun with polyamide 6 as the positive triboelectric material and PVDF nanofibers loaded with MXene sheets as the negative triboelectric material [116]. Each nanofiber mat was fixed on a copper electrode and tested in separation-contact mode. Output voltages between 30 and 56 V were produced when the TENG was activated by body motions such as hand clapping and walking. A moisture-resistant stretchable TENG was formed by bonding a PDMS porous layer on a conductive spandex fabric on which silver nanoparticle-decorated rGO nanosheets are bonded with polydopamine [117]. The conductive spandex fabric composite was made superhydrophobic through modification with hexadecanethiol. The output of the resulting TENG was 135 V and 7.5 μA under a 20 N force at 15 Hz. It sustained 15,000 cycles without a change in performance and its energy harvesting performance were fully recovered 37.6 s after being sprayed with water. In addition, the TENG maintained its performance in an environment at up to 80% RH.

A way to enhance the output of textile triboelectric nanogenerators is based on the combination of a narrow-gap textile triboelectric material system with a high-voltage diode and a switch [118]. The narrow-gap system used is a sandwich structure composed of conductive textiles covered with electroactive materials laying face to face, silicone rubber on one side and nitrile rubber on the other side. The charge transfer is controlled by the diode and the activation of the switch, which allows an instantaneous discharging process. Another strategy explored relies on harvesting simultaneously the energy from different sources such as human motion, wind, and rain drops that have different amplitudes and frequencies [119]. The system developed combines a free-standing mode coplanar structure to capture rain drop kinetic energy and a contact–separation mode structure to harvest the mechanical energy from human motion, wind, and rain drops. The free-standing mode structure includes two interdigitated nickel electrodes deposited on a polyester fabric and coated with a parylene film. The contact–separation mode structure consists of a nickel-coated polyester fabric separated with a cotton fabric from a nickel-coated polyester fabric further coated with a parylene film. The free-standing mode and contact–separation mode structures are assembled by sewing. The dual-mode TENG successfully harvested the energy of water drops in sliding and contact–separation mode, as well of the energy associated with air flow and cyclic impact. The researchers also tested its ability to sustain bending and water immersion. In another study, researchers explored combining four functional layers, a triboelectric layer, a piezoelectric layer and two electrode layers, to generate two triboelectric processes and one piezoelectric process during the work sequence of a TENG fabric [120]. The three types of functional layers used silicone rubber as base material: it was blended with PTFE powder in the triboelectric layer, with PZT powder in the piezoelectric layer, and with silver-coated microspheres for the electrode layers. The triboelectric layer was on the top, separated from the piezoelectric layer by an electrode layer. The addition of the piezoelectric layer to the triboelectric system increased the output voltage from 90 to 125 V. The output voltage further increased to 325 V when the PTFE/silicone rubber was replaced by denim fabric in the piezoelectric-enhanced triboelectric nanogenerator.

Efforts have also been dedicated to improving TENG electrodes. For instance, a core/shell organogel electrode was developed to preserve the flexibility and stretchability of textile TENGs [121]. The polymer swollen with an ionic solvent is contained within the core of a silicone hollow fiber, which also provides the piezoelectric activity. The core/shell structure was used to prepare a knitted fabric and tested against electron positive materials such as nitrile rubber. An output voltage of 57 V was measured under an applied force of 100 N. The performance was maintained after 100 cycles of bending and 200% stretching, as well as when exposed to temperatures as low as −20 °C and as high as 45 °C. Moving from the traditional series configuration to a parallel electrode connection was shown to allow compensating for the higher resistance of stretchable electrodes compared to metal electrodes [122]. A plain weave fabric prepared using a core/shell organogel electrode with the different yarns being connected in parallel led to a decrease in the inner resistance by an order of magnitude and an 11.8-time increase in the output current compared to a knitted fabric using the same core/shell organogel electrode. Other researchers explored the use of human skin both as one of the triboelectric layer and as the electrode [123]. Voltage of up to several tenths of volts were generated in contact mode with a PTFE film during typical body motions.

## 5. Thermoelectric Energy Harvesting

Work on textile thermoelectric energy harvesting has been limited by the fact that materials showing a large Seebeck effect, i.e., bismuth telluride (Bi_2_Te_3_) and antimony telluride (Sb_2_Te_3_), are brittle and do not allow an easy integration into textile structures [94]. Researchers have also explored the use of doped conjugated polymers such as PEDOT: PSS and carbonaceous materials to prepare flexible thermoelectric modules [124]. Progress in these two areas is described below.

As a complement to thermoelectric energy harvesting systems, a textile heat flow sensor was formed by inserting a constantan-based bimetallic wire into a textile structure during the weaving process [125]. The thermocouple junctions on both faces of the fabric were prepared by electrolytic deposition of copper on the constantan wire after local coating with a resin, which was later removed. The fabric is permeable to water vapor, which allows moisture transfer to be considered when measuring heat exchanges between the human body and its environment.

### 5.1. Flexible Inorganic Thermoelectric Modules

A telluride thin film was prepared by electrodeposition, then transferred to a flexible substrate [126]. The film had a high flexibility as well as a Seebeck coefficient of 356 μV K^−1^ and a power factor of 3.21 μW cm^−1^ K^−2^ at room temperature. The authors suggest that the same technique could be used to prepare thin films of Bi_2_Te_3_ among others. Bi_2_Te_3_ powder was also combined with a polymer and a solvent to prepare flexible thermoelectric films by electrospinning [127]. The use of PVA as the polymer led to 150–600 nm diameter fibers decorated with Bi_2_Te_3_ particles along their length. The electrospun film displayed a Seebeck coefficient of −10.6 mV K^−1^. Improvements in the electrical conductivity could be achieved with a conductive polymer such as PEDOT: PSS. Another strategy involves the growth of inorganic compounds on textile surfaces. For instance, Lu et al. synthesized Bi_2_Te_3_ and Sb_2_Te_3_ nanocolumns on both sides of a silk fabric [128]. The p-type and n-type thermoelectric nanocolumns were connected on both sides of the fabric using silver foil and silver paste. With an array of 12 of such thermocouple junctions, a power output of 15 nW was recorded when a temperature difference of 35 K was applied between both sides of the fabric. Ikeda et al. synthesized Sb-ZnO and Ag-ZnO nanostructures on a cotton fabric by a solvothermal process [129]. The Sb-ZnO nanostructures showed a negative Seebeck coefficient in the range of 200 μV K^−1^ and the Ag-ZnO nanostructures a positive Seebeck coefficient also in the range of 200 μV K^−1^, opening the door to the fabrication of a flexible thermoelectric power generation module.

**Figure 9:**
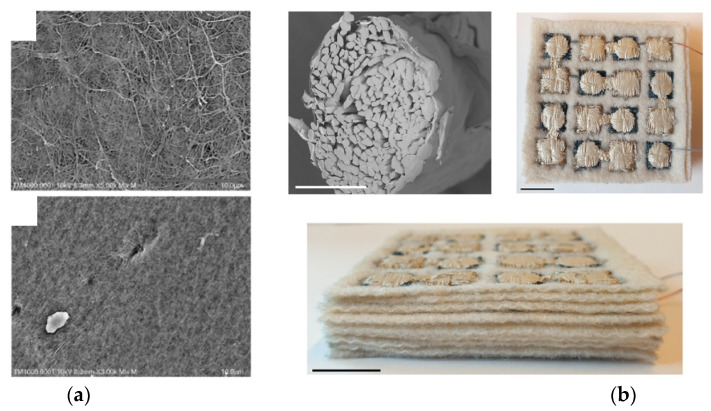
Examples of recent developments toward thermoelectric energy harvesting materials and structures for textile application: (**a**) MWCNT film before and after doping with PEI, used respectively as the p- and n-type thermoelectric legs (reproduced from [132] with permission from ACS Publications); (**b**) DMSO/PEDOT: PSS coated silk yarn used to prepare an out-of-plane thermoelectric device (reproduced from [138], available through the Creative Commons license (creativecommons.org/licenses/by/4.0/)).

N-type and p-type thermoelectric materials were also prepared using single-wall carbon nanotubes (SWCNT) [130]. Thin films composed of doped SWCNTs were formed by solution processing followed by the complete solid state removal of the polymer. Thermoelectric power factors larger than 700 μW cm^−1^ K^−2^ were obtained both for the n-type and the p-type thin films. The SWCNT thermal conductivity decreased with a decrease in diameter, leading to a value of zT of more than 0.1. SWCNTs were also combined with polymers to form flexible n-type and p-type thermoelectric composite films [131]. The p-type thermoelectric materials were formed by dispersing SWCNTs in PDOT: PSS. The n-type films contained SWCNTs doped by polyethyleneimine (PEI); they were encapsulated to prevent the loss of the n-type characteristic in air. A flexible thermoelectric power generator composed of six p-n junctions assembled on a polyimide substrate generated 220 nW when a 50 K temperature gradient was applied. In another study, n-type thermoelectric multiwall carbon nanotube (MWCNT) films were prepared by doping with PEI (Figure 9a) [132]. Pristine MWCNT films were used as the p-type thermoelectric component or leg. Thermoelectric powers of 340 and 520 μW m^−1^ K^−2^ and figures of merit of 0.019 and 0.015, were obtained for the p-type and n-type films, respectively. A p-n segmented thermoelectric yarn was prepared using a CNT yarn bearing PEDOT: PSS and PEI-impregnated segments separated by pristine CNT segments [133]. The power factor was 512.8 μW cm^−1^ K^−2^ for the PEDOT: PSS/CNT yarn and 667.8 μW cm^−1^ K^−2^ for the PEI/CNT yarn. The segmented thermoelectric CNT yarn was used to prepare a warp knitted spacer fabric without losing its performance during the manufacturing process. A power output of 51.5 mW m^−2^ was achieved under a temperature gradient of 47.5K. When the thermoelectric spacer fabric was placed in contact with bare human skin in an environment at 22 °C, an output voltage and a power of 0.62 V and 10.12 mW, respectively, were obtained, which is sufficient to power gas sensors according to the authors of the study. Finally, the Seebeck coefficient of thermocouple junctions formed using nickel-coated carbon fiber yarns, both original and after etching in a mixture of hydrochloric acid and hydrogen peroxide, was measured when subjected to a temperature gradient between 0 and 25 K [134]. The values, which ranged between 0.5 and 18 μV K^−1^ depending on the level of etching, were successfully described by a simple model based on the yarn linear electrical resistance.

### 5.2. Organic Materials/Textiles

Various conjugated conductive polymers can be used to prepare thermoelectric energy harvesting devices [135]. For instance, polypyrrole was coated on a polyester net-type fabric by in situ polymerization during deposition [136]. When combined with a copper wire, it exhibited a Seebeck coefficient of 13.3 μV K^−1^. The advantage of this material is its low thermal conductivity, leading to a high figure of merit, zT. With a PEDOT: PSS coating applied on a cotton nonwoven by solvent evaporation, Kirihara et al. were able to light up an LED with a temperature gradient of 48.5 K using a nickel foil as the second element of the thermoelectric device [137]. The Seebeck coefficient measured was in the range of 20 μV K^−1^. PEDOT: PSS was coated on a silk sewing thread, which was further covered with DMSO (Figure 9b) [138]. The PEDOT: PSS thread was used to prepare an out-of-plane thermoelectric device by embroidery on felted wool fabric using a silver-plated polyamide thread as the second component of the thermoelectric p-n junction. A power of 1.2 μW was produced when the thermoelectric device was exposed to a temperature gradient of 65 K. In another study, PEDOT: PSS fibers were prepared by wet spinning into sulfuric acid for application in thermoelectric energy harvesting [139]. In addition to a thermoelectric power of 30 μW cm^−1^ K^−2^, the semi-crystalline fibers displayed a Young’s modulus of up to 1.9 GPa. The researchers reported that repeated bending and stretching beyond the yield point did not considerably affect the electrical properties of the fibers.

A simple process was used to turn a cotton fabric into a p-type and n-type material usable for thermoelectric energy harvesting [140]. A coating of rGO was formed on the fabric by a succession of pad–dry–cure applications and reductions by L-ascorbic acid. The p-type material was produced by further coating the fabric with PEDOT: PSS. The washing resistance was tested for up to 20 washes and showed a gradual increase in the sheet resistance with the number of washings: the sheet resistance increased by 1.7 times after 20 washes for the rGO-coated fabric and by 3.5 times for the PEDOT: PSS/rGO coated fabric. A 20% rGO coating on the fabric led to a power factor of 150 μW cm^−1^ K^−2^ for the PEDOT: PSS/rGO-coated fabric. A 10-leg thermoelectric device was prepared using the PEDOT: PSS/rGO- and rGO-coated fabrics assembled in a parallel arrangement with copper tape and conductive thread. Finally, a thermoelectric wristband was formed using a polyacrylonitrile-silica nanofibrous film prepared by electrospinning [141]. Coating this nanofibrous film with PEDOT: p-toluenesulfonic acid via low-temperature in situ interfacial polymerization yielded the formation of coral-like features on the nanofiber surface. This p-type material achieved a conductivity of 24.5 S cm^−1^ and a Seebeck coefficient of 13.67 μV K^−1^. The n-type material involved a coating of the nanofibrous film with silver nanoparticles by wet electroless deposition, with a conductivity of 100 S cm^−1^. The wristband formed with seven p–n legs exhibited an output voltage of 0.18 mV under a temperature difference of 10 °C between the skin and the environment.

## 6. Current Challenges and Perspectives on Promising Avenues of Further Development

Energy harvesting systems are critical to allow the development of a solid and sustainable wearable electronics and smart/e-textile industry. Several products have reached the market. Most of them are based on photovoltaic systems and use the inorganic semiconductor technology. This includes photovoltaic canvas for tents, shades, and canopies from Tarpon Solar, Norway, in partnership with Midsummer, Sweden [142], a solar shirt by Pauline van Dongen [143], the solar jacket from Tommy Hilfiger [144], and the solar purse from Noon Solar bags [145]. Products based on piezoelectric energy harvesting include an orthopedic prosthetic vest developed in Germany [146], the Power Shorts from Vodafone [147], and a dance-powered phone charger [148]. In terms of thermoelectric energy harvesting, commercial products include a sleeping bag by Vodaphone [149], and rubber boots by Orange [150].

Table 1 provides a list of strengths and weaknesses of photovoltaic, piezoelectric, triboelectric, and thermoelectric energy harvesting within the context of smart/e-textiles application. If large progress has been made over the last few years and is still occurring at a rapid pace as evidenced by the large number of scientific articles recently published, the technologies behind wearable energy harvesting have not reached maturity yet and many challenges remain [151,152,153,154,155]. In general, technologies with high energy conversion efficiencies lack the flexibility and low weight required for smart/e-textile applications. In addition, many smart/e-textile-relevant technologies suffer from robustness, reliability, and durability issues, including with washing, bending, abrasion, contact with water and other liquids, etc., and may not be able to sustain the stress applied during textile manufacturing and while in use. Technologies developed also need to preserve the textile aspects of the product, including its flexibility, drapability, touch, breathability, heat and moisture management, etc. In addition, manufacturing processes need to be scaled up to industry production levels in a cost-efficient manner. Finally, concerns exist on the possible impact of certain compounds on health and the environment, especially in a context of clothing and other general consumer textile products, which will probably be landfilled at the end of their life. No attempt was made in Table 1 to provide a rating of the different technologies or materials due to the numerous developments underway and the fact that progress is made on several fronts, including materials as well as structures at the macro/micro/nano scale and processes.

Strategies proposed to help solving some of the challenges mentioned can be grouped in three categories: technical, interdisciplinary collaborations, and development of dedicated test methods. Table 1 identifies promising technical strategies. They include producing multiscale and hierarchical structures, engineering matter at the nanoscale, combining different types of materials and different actuation methods, using core/shell structures for encapsulation, and controlling precisely the manufacturing process. For instance, higher energy conversion efficiencies can be obtained by combining PENG and TENG devices within the same textile structure [92]. Energy harvesting systems may also comprise a storage unit [156]. They may have a dual use as a strain sensor [88] or a motion sensor [73] in the case of piezoelectric systems, or as a haptic sensor [117] or a motion sensor [116] in the case of triboelectric systems.

In addition, as textiles are complex materials and the challenges faced to develop textile-friendly energy harvesting systems are multidisciplinary, a truly interdisciplinary approach is required, bringing together the relevant expertise: textiles and clothing, design, materials, different fields of engineering, manufacturing, human factors, etc. Starting from the users’ needs and considering the industry capacity, a holistic, human-centered perspective is key to finding sustainable solutions to the energy supply challenge of smart/e-textiles and wearable electronics.

Finally, no significant progress can be achieved in terms of market development unless appropriate and standardized test methods are available to the industry [157]. They are critical to ensure that smart/e-textile manufacturers are able to control the quality of their products in terms of efficiency, safety, and durability. If a standard test method has been developed for the measurement of the linear resistance of conductive tracks for electrically conductive textiles [158] and the existing ASTM D4496 and AATCC TM76 standard test methods can be used to characterize their surface resistance [159], nothing exists yet or is in development to provide a standardized way to assess the performance of textile energy harvesting systems.

## 7. Conclusions

Energy harvesting systems are key to sustainable progress in smart/e-textiles, including sensors. Relevant energy harvesting technologies include photovoltaic, piezoelectric, triboelectric, and thermoelectric. Large progress has been achieved over the last few years toward to the development of flexible systems with a higher power output. Promising strategies include multiscale and hierarchical structures, engineering matter at the nanoscale, combining different types of materials and different actuation methods, using core/shell structures for encapsulation, and controlling precisely the manufacturing process.

However, many challenges remain: power output still insufficient; issues with robustness, reliability, and durability; inability to preserve textile aspects such as flexibility, drapability, touch, breathability, heat and moisture management characteristics; high cost; processes difficult to scale up; and potential toxicity of certain compounds. The key to finding solutions to the multifaceted challenges raised by energy harvesting systems for smart/e-textile application is a holistic, human-centered, and interdisciplinary approach: starting from the users’ needs, bringing together the different relevant expertise, and considering the industry manufacturing capacity. Another critical aspect to tackle is the development of standard test methods for textile energy harvesting systems to ensure that smart/e-textile manufacturers are able to control the quality of their products in terms of efficiency, safety, and durability.

## Figures and Tables

**Table 1:** Strengths and weaknesses of photovoltaic, piezoelectric, triboelectric, and thermoelectric energy harvesting in the context of smart/e-textiles applications, and promising strategies.

Technologies	Strengths	Weaknesses	Promising Strategies
**Photovoltaic**			
Inorganic films	High energy conversion efficiency	Rigid, brittle material; lack textile appearance and behavior; expensive; concerns with toxicity of compounds	Thin films, amorphous silicon, perovskites, tandem structures, combined with polymer layers
Organic films	Flexible, thin, light; can be manufactured using continuous processes with which the textile industry is already familiar	Low energy conversion efficiency	Ternary polymerization, side chain engineering, nanocomposites, surface texturing of substrate
Dye-sensitized solar cells	Low cost, can be manufactured using continuous processes with which the textile industry is already familiar	Low energy conversion efficiency	High purity sensitizers, ionic liquid electrolytes, nanofibrous transparent electrodes
Fibers and filaments	Can be fully integrated in the textile structure	Low energy conversion efficiency, low resistance to bending	Core/shell structures, electrospun fibers
**Piezoelectric**			
Ceramics and single crystals	High output power	Rigid, brittle material; toxicity in certain instances	Use as nanofillers in polymer matrices
Polymers	Flexible, thin, light; Can be easily integrated into textile structures as a film or a yarn	High output power, low durability	Precise control of the manufacturing parameters to optimize the crystallinity and piezo phase content; nanofibrous structures
Composites/ nanocomposites	Flexible, thin, light; Can be easily integrated into textile structures as a film or a yarn	High output power, low durability	Nanofibrous structures, piezoelectric fillers, multiscale features, lead-free alternatives to piezoelectric ceramic fillers
PENG devices	Level of miniaturization desired for smart textiles	High output power, low durability	Coiled fibers, PDMS encapsulation, paired with TENG device
**Triboelectric**			
Films/coatings	Flexible, thin, light; Can be easily integrated into textile structures	Limited power output, sensitive to moisture, low durability	Micro/nanopatterning
Fibers/textiles	Flexible, thin, light; Can be easily integrated into textile structures	Limited power output, sensitive to moisture, low durability	Micro/nanopatterning, hierarchical structures, electrospun nanofibers, knitted/double-faced knitted structures
TENG devices	Level of miniaturization desired for smart textiles	Limited power output, sensitive to moisture, low durability	Combination of charge flow paths, core/shell organogel electrode, combination of actuation modes, paired with PENG device
**Thermoelectric**			
Large Seebeck effect materials	High energy conversion efficiency	Poor processability, toxicity, high cost	Use as nanofillers in conductive polymer matrices, nanoscale structures
Carbonaceous materials	Easy to turn into p- and n-type materials by doping, easy to integrate into textile structures	Low durability	Combination with conductive polymers, yarn configuration for out-of-plane thermoelectric devices
Organic materials/textiles	Flexible, thin, light; low thermal conductivity	Low energy conversion efficiency, low durability	Nanoscale structure (nanocomposite, nanofibers, etc.), out-of-plane construction

## Data Availability

As this is a review paper, data is available in the articles cited.
